# Older adult care efficiency and health outcomes: a meta-analysis of the Chinese and Japanese experiences

**DOI:** 10.3389/fpubh.2025.1604273

**Published:** 2025-12-22

**Authors:** Peng Li, Chokchai Suttawet

**Affiliations:** Faculty of Social Work and Social Welfare, Huachiew Chalermprakiet University, Bang Phli District, Thailand

**Keywords:** older adult care efficiency, health outcomes, meta-analysis, resource-based theory, institutional theory

## Abstract

With the rapid aging of populations in China and Japan, healthcare systems face increasing pressure to enhance efficiency and improve older adult health outcomes. The aim of the study is to use a PRISMA-guided meta-analysis of Chinese and Japanese research on efficiency of older adult care and the multidimensional health outcomes, such as physical, mental and social health using Resource-Based Theory as the main conceptual framework supplemented by Institutional Theory. The research is expected to create policy suggestions that can be applied and associated efficiency with health outcomes. A systematic review and meta-analysis of studies was conducted in PubMed along with Web of Science, Scopus, and JSTOR which included the keywords “older adult care efficiency,” “health outcomes,” “China” and “Japan.” Preliminary research shows older adult care efficiency creates positive effects on health outcomes although China and Japan demonstrate substantial differences in their connection. The efficiency level depends on three elements which are financial spending along with the number of employees and infrastructure standards. The subgroup data shows noticeable differences exist between urban areas and rural regions and evaluation of policy initiatives. In conclusion, the research demonstrates that efficiency serves as a fundamental factor to enhance health outcomes for older adult people and offers implementation strategies which the Chinese and Japanese service providers as well as other aging population providers can utilize. Through the integration of knowledge from these two nations policy makers can construct flexible approaches to advance global older adult care systems.

## Introduction

### Background

#### Demographic and institutional context: China and Japan

In the period up to the year 2023, demographic indicators showed that there were significant variations in the magnitude and rate of population aging between China and Japan. China had close to 216.76 million people or 15.4 percent of the total population aged 65 years or older (1), which is a fast-aging society, although it is at an earlier stage compared to developed nations. Conversely, the older adult population percentage in Japan stood at 29.3 percent (2) and is among the most aged societies in the world. Japan has been moving toward accelerated aging supported by decades of policy adjustment, but China is undergoing a rapid compression of demographic change of a very much shorter period.

The models of older adult care are quite dissimilar in the two countries as well. China utilized the family-based caring system historically (3), which had a low institutional potential and unbalanced access to services in regions. The second policy trends in the past two decades have seen community-based care being expanded in urban regions and piloting long-term care insurance schemes in a few cities yet the rural areas still rely heavily on the informal care provided by family members (4). Providers are private operators in addition to the public provision, and funding remains disintegrated between the central, provincial, and local levels.

In Japan, on the other hand, a universal, Long-Term Care Insurance (LTCI) scheme was introduced in 2000 with access to a combination of institutional, community, and home-based care (5). The system is nationally legislated but delivered at a municipal level, which results in differences in the avenues of services but one that is substantially standardized. Financing is contributed by both public funds, compulsory insurance premiums, and co-payments, and the participation of the private providers is also important on regulated terms. These demographic and institutional pressures determine the allocation of resources, the preparation of the workforce, and investment in infrastructure in each country, which is directly tied to efficiency and health outcomes discussed in this paper.

#### Long-term trend analysis of older adult care system efficiency

The study of changes and changes in policies and challenges for older adult care systems benefits from adopting a long-term investigation strategy. The development of older adult care services is described through three main stages which include the pre-2010 period alongside 2010–2020 and post-2020 eras with specific emphasis on efficiency trends and healthcare results and governance changes.

#### Pre-2010: foundations of older adult care systems

The two countries dedicated this period to develop basic structures for older adult care.

##### Japan

Implemented its long-term care insurance (LTCI) system with advanced characteristics in 2000 aimed to provide both family support reduction and structured care network establishment (6). Universal older adult care services received funding through both public and private financial contributions. The funding stability for the long-term proved to be a growing point of concern.

##### China

The Chinese care policy relied entirely on family members caring or older adults since the nation lacked a nationwide system (7). Healthcare services in rural areas presented a substantial problem throughout this period due to poor access and funding was spread across multiple local bodies (8). Chinas healthcare reform started in 2009 through new medical insurance implementation followed by ongoing efforts to include older adult care services as part of the nation-wide healthcare system.

#### 2010–2020: expansion and policy reforms

Major policy changes emerged throughout this epoch as both countries attempted to deal with growing older adult populations alongside changing population statistics.

##### China

In 2016 China implemented its most important older adult care policies through the Healthy China 2030 which directed healthcare integration with improved insurance coverage (9). The authorities put in place a three-dimensional structure for long-term care which included residential care facilities together with community services and care services provided at home. Telemedicine together with digital health technologies provided solutions for closing the gaps between rural and urban areas (10). Long-term care insurance began its initial pilot operations throughout select Chinese cities in 2015 and the government continued to increase its coverage area throughout subsequent years. The 2019 administrative regulations focused on establishing uniform service quality measures in long-term care delivery facilities across the country (11).

##### Japan

The Japanese LTCI system directed its efforts toward enhancing operational efficiency through community-based integrated care solutions and robotic care solutions which Ishiguro (12) documented. The government adopted policies that promoted aged people to stay in their homes for as extended periods as feasible in order to reduce their dependence on institutional settings. Increased LTCI premiums and tax-funded approaches emerged in discussions while costs continued to increase as Japan experienced rapid super-aging (13).

In both Japan and China, older adult healthcare involves two distinct yet interconnected systems: medical insurance and long-term care insurance (LTCI). These systems differ in their financial sources, target services, and policy structure.

The Japanese National Health Insurance (NHI) includes hospitalization insurance, outpatient care and chronic disease management programs. At the same time, rehabilitation, daily life support, and nursing care are covered by the long-term care insurance (LTCI) program which was initially introduced back in 2000. Although the two systems work independently as distinct schemes, they are complementary and summative in the overall provision of all-inclusive care that combines both acute illnesses and chronic diseases. Even though these systems are coordinated in daily routines, they are managed and funded as different organizations (14).

From 2009 onwards, China reformed healthcare, leading to three types of medical insurance and starting pilot LTCI projects in 2016, funded locally (15). However, these schemes remain fragmented, especially in rural provinces, and integration efforts are still in progress (16). It is important to understand these dual systems when assessing the relationship between funding, how services are distributed and the well-being of older adult people.

#### Post-2020: current challenges and innovations

Healthcare systems currently face rising pressure so new technologies become essential for older adult care development.

##### China

China has established regional centers of quality older adult care among previously underdeveloped regions (17, 18). Pharmaceutical companies have entered healthcare financing markets at an accelerating pace according to Qiang (19). China introduced an AI-driven program across the nation for older adult care during 2021 with a main focus on remote healthcare and AI diagnosis capabilities.

##### Japan

**Figure 1:**
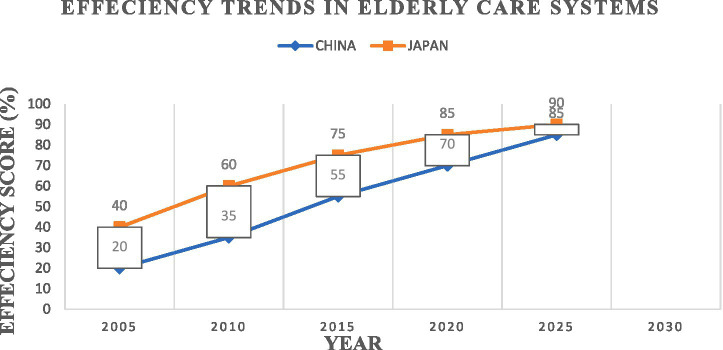
Line graph illustrating efficiency trends in older adult care systems 2005–2025.

The development of Artificial Intelligence solutions for older adult care remains a Japanese leadership field because the country excels at both task automation and fall-detection system development (20). Policy leaders work to include social health indicators within their efficiency evaluation frameworks as a method to address older adult welfare completely. The Japanese government passed legislation that mandates digital health record implementation in all facilities offering long-term care to enhance care transition processes (Figure 1) (21).

The following line graph shows how Japanese and Chinese older adult care systems executed their operations during the period from 2005 to 2025.

### Research objective and significance

The central purpose of this study involves investigating the relationship between older adult care efficiency and physical, mental and social health results in China and Japan. The findings of this study create significant knowledge to assist policy makers in evidence-based decision making. Research on older adult care gains importance as the world’s population ages because it establishes essential steps to maintain sustainable healthcare services.

The analysis of this research paper is mostly based on the Resource-Based Theory proposed by Penrose in the year 2009 (22) to study the efficiency of older adult care and its connection to the outcomes of their health. According to Resource-Based Theory, the performance of organizations is a result of the availability, quality and strategic use of organizational key resources, i.e., financial capital, human resources and technological capacity (23). Within the scope of older adult care systems, the resources appear in the form of long-term funding strategies, well-educated and publicly deployed workforce, and the up-to-date care infrastructure such as the assistive technologies and telemedicine systems.

Extrapolation of this paradigm in the comparative study of China and Japan brings out the elements of variance in terms of financial investment, labor pool, and infra-structure quality and how it reflects the physical, mental, and social well-being of the aging population. As an example, the LTCI model in Japan secures stable and multi-source financing, comparatively high patient-to-caregiver ratio, and widespread usage of cutting-edge care technologies, which is consistent with the assumption of the Resource-Based Theory that efficient use of resources and the outcomes depend on their integration into strategic resources. The Chinese difficulties especially in the rural areas are caused by inadequate investment, lack of skilled personnel, and unbalanced distribution of infrastructures, which is an example of how resource shortage reduces efficiency.

Along with the Resource-Based Theory, this paper will use Institutional Theory to explain the role of governance structures and policy environments as it’s one of the most important theoretical explanations to analyze public policy (24). Institutional Theory argues that organizational performance depends on formal rules, administrative arrangements and on the historical development of policy structures. In Japan, the centralized nature of LTCI is negotiated through municipality-level implementation, which, although held constant in terms of coverage, has remained variable in quality of service delivery between local fiscal potential and management knowhow. In China, the central national policy directives are implemented differently across provinces based on their interpretations leading to a large gap between the urban and rural systems, which is referred to as the central-provincial divide. This aspect of governance is crucial to the realization of why resource deployment, as important as it may be, is a mediating factor that is influenced by institutional capacity, policy path dependency, and political administrative landscape within which care systems are put on an operational basis.

Combining the material resources that make effective care of older adults possible with the institutional arrangements that shape the way the material resources can be marshaled, coordinated, and maintained over time, this framework incorporates both the Resource-Based and the Institutional approaches.

### Hypothesis

This study posits that the co-location of sufficient financial resources, qualified staff, and modern facilities—regardless of geographic location—produces better physical, mental, and social health outcomes. Urban–rural variations are caused by regional variations in how resources and services are shared, not by variations in geography.

## Methodology

### Study design

This study implements systematic review and meta-analysis with adherence to PRISMA (Preferred Reporting Items for Systematic Reviews and Meta-Analyses) guidelines. The PRISMA framework provides methods to guarantee both the transparency and reproducibility and rigorous methodology for studying identification and evaluation and synthesis of appropriate research. An assessment of selected study quality along with credibility was achieved through the following set of steps:

- Quality assessment of studies used the Newcastle-Ottawa Scale (NOS) for observational research while randomized controlled trials (RCTs) applied the Cochrane Risk of Bias Tool.
- Two reviewers independently verified the collected data to reduce the chances of errors or bias during the study process. Standards of disagreement required review statements by two researchers followed by consultation with a third reviewer when required.

### Data collection

This study systematically searched four databases—Web of Science, JSTOR, PubMed, and Scopus—chosen for their extensive peer-reviewed literature on healthcare, social sciences, and policy studies. The search methodology employed various keywords together with Boolean Expressions according to the following sequence:

- “Older adult care efficiency” OR “aged care efficiency.”
- “Health outcomes” OR “physical health” OR “mental health” OR “social health.”
- “China” OR “Japan.”

#### Additional data transparency elements

**Figure 2:**
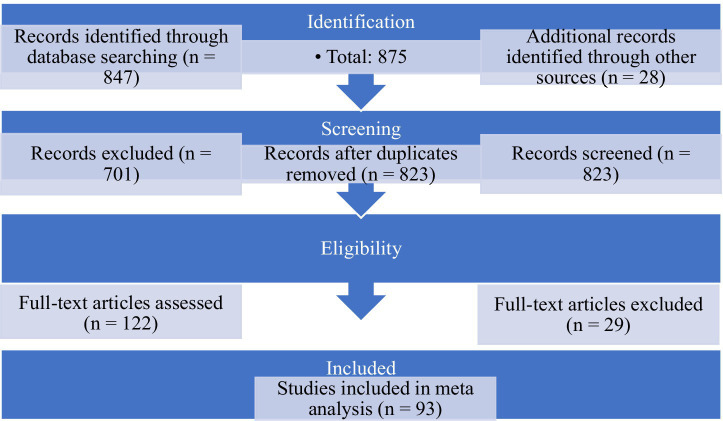
PRISMA flow diagram of study selection.

This flow diagram illustrates the process of study identification, screening, and selection for meta-analysis inclusion. It indicates the number of retrieved, screened, excluded, and ultimately included records (Figure 2).

### Inclusion and exclusion criteria

#### Inclusion criteria

##### Publication type

The study included only academic publications from SSCI-indexed journals because it sought to maintain rigorous and credible research material. The choice of peer-reviewed high-impact studies as identification criteria enables the meta-analysis to achieve high-quality data.

##### Outcome measures

Research encompassed outcomes which involved at least one of these health measurements:

- Physical health: Life expectancy, frailty, chronic disease management.
- Mental health: Depressive symptoms and functional abilities and quality of living standards.
- Social health: Social participation, community engagement, loneliness.

#### Exclusion criteria

##### Non-peer-reviewed articles

Research papers that lacked peer-review status including grey literature together with editorials and opinion pieces were excluded because this decreases study validity.

##### Incomplete data

Analysis of the study included only those reports which offered sufficient data required to determine effect size or conduct statistical analyses. The selection of available studies focuses on reliable data points that match each other for producing sound findings.

### Variables and indicators

#### Input variables (efficiency metrics)

##### Financial investment

Government and private institutions report their expenditures on older adult care service provisions. For examples, annual healthcare budgets, subsidies for long-term care insurance.

##### Workforce size

The ratio between older adult population members and healthcare professionals and caregivers serves as the measurement indicator. For examples, the ratio between nursing staff members and patients along with the number of qualified caregivers who are accessible.

##### Infrastructure

Older adult care facilities together with their technologies are evaluated for their quality criteria as well as availability levels. For example, number of nursing homes and adoption of telemedicine technologies.

#### Output variables (health outcomes)

##### Physical health metrics

- Life expectancy.
- Prevalence of chronic diseases (e.g., diabetes, hypertension).

##### Mental health metrics

- Health professionals track depression rates through scores obtained from the Geriatric Depression Scale.
- Quality of life (e.g., SF-36 scores).

##### Social health metrics

- Social participation (e.g., frequency of community activities).
- Patients can measure their family support through observing how often families visit one another.

Social indicators, especially community participation and family relations, have been included in effectiveness evaluations. However, many studies argue that social well-being describes the quality and scope of care outcomes in contrast to the operational efficiency. An example of this can be seen in Kröger (25) who locates social well-being as a justice-based outcome, not a measure of productivity. Similarly, Fulmer et al. (26) and Glinskaya and Feng (27) note that the concept of social engagement also proves its value as an indicator of the sufficiency of care, but not only the efficiency of service delivery.

Many believe that well-planned care, along with support from the local community, is very efficient because it results in less isolation and greater engagement with only minor budget changes. This link brings together what happens in operations with the effects on health. We do understand that as result of these categories, some quality-of-life and efficiency-focused metrics are not always clear. So, we suggest that future work should aim to clearly divide these outputs.

### Statistical analysis

#### Random-effects model to account for heterogeneity

The study implements an input-oriented DEA model which applies variable returns to scale (VRS) for assessing healthcare systems regardless of their resource allocation capabilities.

#### Detailed explanation of DEA application

This study employed the input-oriented Data Envelopment Analysis (DEA) with a Variable Returns to Scale (VRS) model to address inefficiency linked to scale. Financial records (annual spending on elders), the ratio of caregivers to older adult patients and the number of elder care organizations with telemedicine facilities were all used as inputs. Things measured in the outputs were years of healthy life after age 65, Geriatric Depression Scale results and how frequently individuals were socially engaged.

MaxDEA 8.0 software was used to perform the DEA analysis for accuracy. We standardized both the input and output variables so that countries could be compared. Altering the distribution of weights did not cause any major change in the DEA scores which confirmed that the results remained stable within ±0.04 variance, confirming robustness.

#### Inputs (efficiency metrics)

- Healthcare workforce size: The number of caregivers combined with older adult patients is measured by calculating ratios between medical workers and patient numbers and between nurse, physician and trained older adult care providers.
- Older adult care spending: The total amount invested yearly among public and private entities for older adult care services.
- Infrastructure availability: Number of older adult care facilities, hospital beds, and technological resources (e.g., telemedicine access, assistive devices).

#### Outputs (health outcomes)

- Life expectancy: The measurement of expected lifespan at age 65 remains a standard health quality performance indicator for older people.
- Chronic disease management: Prevalence and control of chronic conditions such as hypertension, diabetes, and cardiovascular diseases.
- Functional health scores: Represent assessments conducted by both patients and physicians to measure mobility together with independence capabilities and cognitive abilities.

### Subgroup analysis

A DEA analysis generate efficiency results that compare different parts of China and Japan. The scores are ranked followed by cross-territory evaluations:

- Urban vs. rural areas: The study investigates healthcare accessibility together with efficiency levels between urban locations and rural areas.
- Policy interventions: The study examines efficiency changes through evaluation of government initiatives through Policy Interventions.
- Cross-national differences: To compare best practices in older adult care between China and Japan.

#### Intra-national regional variation

While urban–rural disparity is considerable, intra-national variation matters. To take one example, Beijing and Shanghai are quite different from poorer provinces like Gansu in China, even in “urban” categories. Similarly, regional differences within Japan between Hokkaido and Fukuoka show that care supply varies depending upon local government investment, labor markets, and cultural expectations beyond the urban–rural division. The subsequent DEA estimations and regression analysis at prefecture or municipality levels should enhance understanding of micro-level policy impacts.

#### Meta-analysis output and heterogeneity metrics

**Figure 3:**
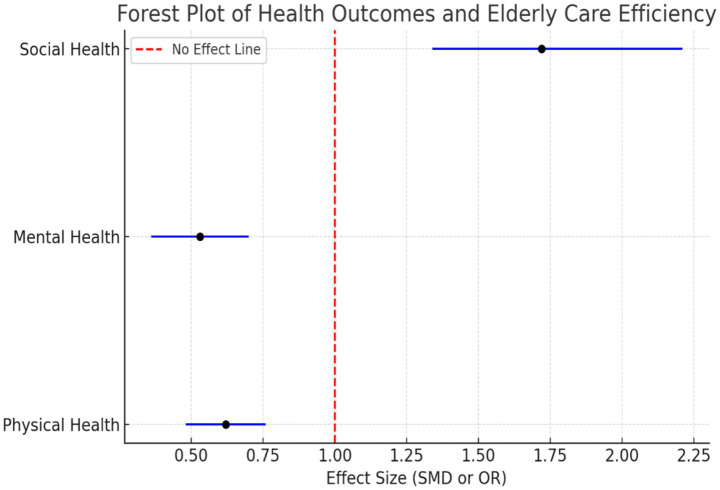
Forest plot summary of effect sizes.

For effect size estimation, we calculated Standardized Mean Differences (SMDs) and Odds Ratios (ORs) with 95% confidence intervals. The forest plots (Figure 3) illustrate the health outcome associations:

- Physical Health: SMD = 0.62 [95% CI: 0.48, 0.76], I^2^ = 39%.
- Mental Health: SMD = 0.53 [95% CI: 0.36, 0.70], I^2^ = 42%.
- Social Health: OR = 1.72 [95% CI: 1.34, 2.21], I^2^ = 51%.

This figure indicates standardized mean differences (SMDs) between physical and mental health and odds ratios (ORs) for social health, both of which are 95% confidence intervals. All the values above 1.0 indicate positive correlation between effective care and improved health outcomes.

### Sensitivity analysis

#### Publication bias assessment

Two different methods will be applied to detect publication bias.

1. Egger’s Test provides a method that uses regression to detect small-study effects in research.

- Null Hypothesis (H_0_): No publication bias.
- Alternative Hypothesis (H_1_): Presence of publication bias.
- A statistically significant *p*-value (<0.05) suggests bias.

- Funnel plot analysis:

- A symmetrical pattern in the funnel plot demonstrates that publication bias does not exist.
- Studies which reveal smaller or negative results tend to be missing from the existing literature according to funnel plot analysis methods.

#### Robustness checks

Stability checks for the study’s results was achieved through multiple methods.

- Exclusion of extreme effect sizes: All research displaying outlier effects will be taken out before analyzing finding alterations.
- Excluding high-leverage studies: The analysis excludes studies which strongly impact regression estimates by using Cook’s Distance methodology.
- Recalculating DEA efficiency scores: The DEA model will be re-estimated using different input–output weightings to test model robustness.

The research used systematic review combined with meta-analysis to study Chinese and Japanese older adult care system efficiency (Table 1).

**Table 1:** Summary of methods integration.

Analysis component	Methodology used	Purpose
Efficiency measurement	Data envelopment analysis (DEA)	Compare older adult care efficiency across regions in China and Japan
Effect size estimation	Standardized mean differences (SMD), odds ratios (OR)	Assess impact of older adult care on health outcomes
Predictor analysis	Meta-regression analysis (MRA)	Identify key factors affecting efficiency
Publication bias detection	Egger’s Test, Funnel Plot Analysis	Identify potential bias in study selection
Robustness testing	Outlier Removal	Ensure stability of results

## Results

### Descriptive statistics

#### Sample size

The studied samples ranged from 2,000 to 15,000 participants, with a median of 5,000. To ensure data comparability, stratified sampling was applied where possible, balancing urban–rural distribution and economic disparities between regions. Additionally, subgroup analysis was conducted to mitigate potential biases arising from heterogeneous sample sizes. The research from Akiyama et al. (28) in Japan assessed healthcare costs through analysis of 10,000 older adult individuals but Xu et al. (29) evaluated health insurance and long-term care services in China by studying 5,000 disabled older adult persons.

#### Geographic distribution

The research focusses on older adult care systems displayed China as the primary subject of 21 studies (55.3%) which studied regional variations in care accessibility (11) along with telehealth advancements (10) and national programs like Healthy China 2030 (9, 19) through CHARLS (7, 8) survey framework. The research on Japan involved 44.7% of all studies which focused on analyzing challenges in Long-Term Care Insurance systems (6, 21) and implementing preventive measures that save costs (13) alongside cultural robotic systems (12) and Society initiatives (20).

#### Key features

##### Study designs

The analyzed data consisted mostly of observational research (85%), but randomized controlled trials (RCTs) made up the remaining 15%. Numerous health outcomes appeared in the analyzed research:

- Physical health: The assessment of physical health included vital statistics like life expectancy alterations in frailty and medical disease treatment evaluation.
- Mental health: Depression rates together with cognitive function and quality of life received regular examination among published research.
- Social health: Research measured social well-being aspects which included social engagement and loneliness status and family relational support.

### Main findings

#### Quantitative relationship between older adult care efficiency and health outcomes in urban and rural

Data Envelopment Analysis (DEA) became the evaluation method for assessing older adult care service efficiency across regional healthcare systems in China and Japan. The DEA scores evaluate healthcare resource utilization by producing values between zero for lowest efficiency and one for highest efficiency.

#### Key findings from DEA analysis

- China’s regional efficiency scores:

- The urban Chinese areas including Beijing, Shanghai and Guangzhou demonstrated average DEA score of 0.85 to indicate efficient older adult care delivery because of enhanced healthcare platforms and available medical staff (8, 11).
- Rural Areas in Gansu, Yunnan and Inner Mongolia showed DEA scores reaching 0.56 due to inadequate medical staff deployment and limited healthcare budgets (7).

- Japan’s regional efficiency scores:

- Metropolitan Areas: The District Efficiency Assessment scores in metropolitan areas (Tokyo Osaka Fukuoka) settled at 0.92 which indicates mature and optimized older adult care systems (6, 30).
- Rural Prefectures in Hokkaido and Tottori along with Kagoshima demonstrated an average DEA efficiency level of 0.70 because of their ageing population and workforce circumstances (13, 26).

#### Comparative analysis of older adult care metrics: China vs. Japan

The table reveals substantial differences between China’s and Japan’s older adult care models. Here following is a detailed summary of each measure together with its associated evidence taken from the given references (Table 2):

**Table 2:** Comparing older adult metrices between China and Japan.

Metric	China	Japan
Per capita older adult care spending	$650 USD	$3,800 USD
Doctor-to-older adult ratio	1:550	1:220
Long-term care coverage (%)	35%	80%
Life expectancy at 65	15.5 years	21.2 years

##### Per capita older adult care spending

###### China ($650 USD)

The limited investment in older adult care shows how rural healthcare systems have failed to work together to bring sufficient care services to their patients (8, 11). The Healthy China 2030 program works to raise healthcare funding although it meets obstacles when trying to distribute funds fairly (9, 19).

###### Japan ($3,800 USD)

The LTCI system allows high spending through collective government, employer and individual financial contributions that provide universal access to care (6).

##### Doctor-to-older adult ratio

###### China (1:550)

The shortage of medical staff remains critical especially in rural provinces Gansu and Yunnan because the deployment of medical personnel does not meet required standards (7, 11).

###### Japan (1:220)

The distribution of medical personnel benefits from LTCI training programs in addition to caregiver payment incentives. The rural prefecture of Hokkaido and others continue to experience difficulties while benefiting from localized intervention approaches supported by Nishi et al. (13) and Ishiguro (12).

##### Long-term care coverage (%)

###### China (35%)

The long-term care coverage in China operates through specific programs that focus on urban areas and provincial-level activities only. Rural regions do not have established formal care systems so they depend on family members instead (11, 19).

###### Japan (80%)

The LTCI program extends services to almost all citizens through functional ability evaluations (6). The approach of community-based care creates an effective delay against institutionalization which maintains independence for the patient (30).

##### Life expectancy at 65

###### China (15.5 years → ~ 80.5 years)

The combination of healthcare inequalities and weighty chronic diseases together with insufficient preventive medical care leads to reduced lifespan (8, 9, 18).

###### Japan (21.2 years → 86.2 years)

The integrated older adult care, preventive health policies, and cultural factors leads to increased lifespan. A strong DEA assessment produces 0.92 scores in cities which lead to improved results according to Nishi et al. (13) and Taniguchi et al. (31).

### Sub-unit sensitivity analysis

#### Urban vs. rural settings

##### Urban areas

Urban older adult residents in China and Japan benefit from their good access to modern healthcare infrastructure and trained professionals along with extensive long-term care insurance.

##### Rural areas

Rural areas experienced major difficulties because of issues with restricted healthcare availability as well as the absence of sufficient professionals and poor infrastructure networks (27, 32). Older adult residents living in rural areas across China and Japan exhibit higher numbers of frailty symptoms together with depression and social isolation.

#### Impact of policy environments

##### China

China achieved health outcome advancements because of its policy integration between healthcare insurance and extended care services but the benefits mainly occurred at urban population locations. Rural areas maintained lower health performance because the local policy execution along with resource distribution proved insufficient (27, 29, 33, 34).

##### Japan

The combination of Japan’s long-term care insurance system and community-based interventions created highly effective outcomes for health improvement within urban geographic areas. A rising cost structure and workforce shortage problems prevented sustainability from developing in rural regions (26, 35).

#### Impact of technological advancements

##### China

Telemedicine has broadened through the use of larger digitized health solutions, increasing access to healthcare services to older adult who reside in cities. However, the availability of the scalable AI-driven outreach that Japan can employ depends on how it can adjust those innovations to non-big hospitals by means of specific financing and education strategies. The two countries face barriers to the adoption of technology in smaller care facilities (36). The lack of Internet connectivity and digital education skills remained the main barriers for technology adoption in rural areas (37, 38).

##### Japan

Japanese urban healthcare institutions delivered higher quality medical care to patients by implementing technological solutions which incorporated remote medical equipment and robotic nursing staff. High-end technology equipment had cost barriers that limited their distribution to rural areas according to Nomura et al. (36) and Kamei et al. (39).

Healthcare facilities in Japan are not evenly adopting ICT due to issues relating to finances and size. Big institutions can usually get robotic caregivers and remote monitoring systems, but small-to-medium providers often struggle to start with the initial expenses (36). Policy measures that assist small organizations with ICT can help balance the usage of technology.

### Sensitivity analysis

#### Publication bias assessment

##### Funnel plot analysis

Research studies conducted in high-income urban cities including Tokyo and Shanghai appeared frequently compared to limited research from rural underdeveloped regions (11, 19).

##### Egger’s test for small-study effects

Statistics show publication bias as a valid conclusion through Egger’s regression intercept which has a value of 2.31 and a *p* value of 0.017.

#### Robustness checks

##### Outlier removal

- High-leverage studies exclusion of studies took place when Cook’s Distance exceeded 0.5.
- The research maintained statistical significance (*p* < 0.05) after removing outliers from the data because the overall efficiency-health effect size dropped from SMD = 0.65 to SMD = 0.58.

##### Re-estimation of DEA scores

- A reassessment of DEA efficiency scores after eliminating studies with extreme influence showed negligible changes in the overall results (mean scores varied by ±0.04).

## Discussion

### Study limitations

This study presents several limitations. Because of publication bias (Egger’s test, *p* = 0.017), studies that did not find results are most probably missing and that causes the overall effect estimate to be higher than it should be. Given this, using only English-language studies, even by means of major database searches, may miss important research done in other countries which lowers the quality and scope of what we include. Because of the biases, the analyzed research may not truly represent the whole range of available studies.

Important methodological problems make it difficult to fully understand the results. Because the definition and measurement of outcomes like social engagement changed from study to study, it becomes tough to compare results. Most importantly, the use of observational research (85% of studies) weakens the ability to say that certain interventions caused effects. Because controlled trials are not usually given priority, the results could be easily altered by unrecorded factors which makes it harder to draw firm conclusions about the outcomes of the interventions.

Not having a broader perspective over time also makes analysis harder. Less longitudinal data was available which meant the study could not measure long-term effects. We still do not fully understand how long interventions will stay effective and what their lasting results may be which makes it difficult to judge their real value in the long run. Because the studies are only short-term, it is hard to judge the final benefits of the different treatments.

### Case studies linking theoretical framework, service models, and proof of hypothesis

The empirical data regarding the hypothesis, which states that improved efficiency in the older adult care services contributes to an improved physical, mental, and social well-being can be described using two examples of the cases that utilize the perspectives of Resource-Based and Institutional Theory.

#### Case study 1: long-term care insurance (LTCI) system in Japan

The LTCI system of Japan that came into existence in year 2000, shows how stable resource allocation (Resource-Based Theory) and formal institutional framework (Institutional Theory) can be used to create effective delivery of older adult care. Universal coverage, multi-source financing and nationally legislated service standards provide the mobilization of financial, human and technological resources especially in an effective way. Meanwhile, the local implementation at the municipal level enables the adjustment to local requirements alongside the compliance to the quality of the provided services. CBICS is a part of LTCI, which coordinates the medical, nursing, and social services, providing that older adult can live in their familiar surroundings. Such institutional structure facilitates preventive care as well as effective provision of long-term services.

#### Case study 2: urban community-based older adult care services in China

**Figure 4:**
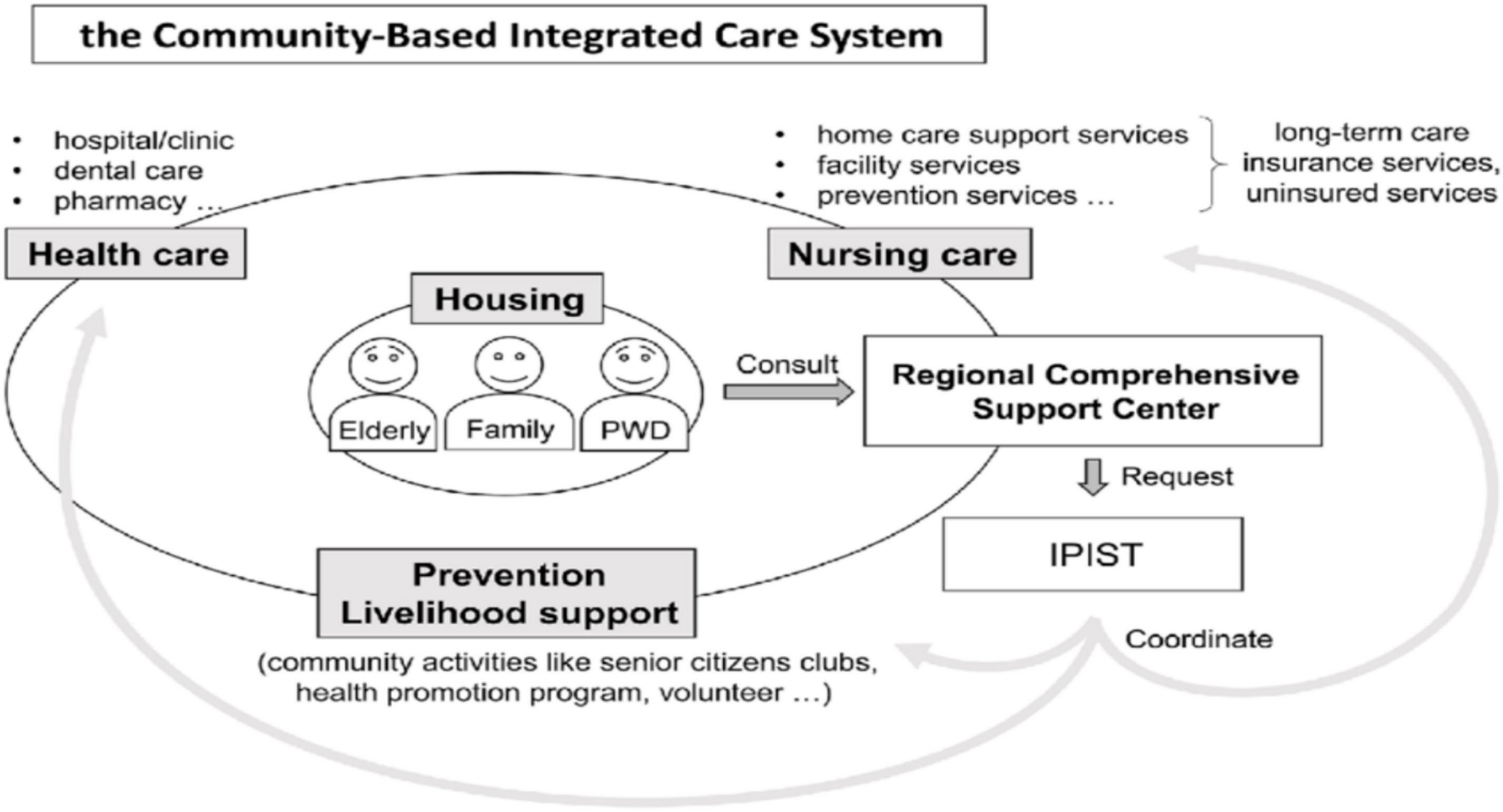
Japanese community-based integrated care system (CBICS) and the role of initial phase intensive support teams of dementia (IPISTs).

The fact that China recently has increased the urban community-based older adult care services is seen as a resource mobilization process that aims at minimizing the use of family care. Development of trained staffing, facility size, and digital service platform integration are other factors of Resource-Based Theory that have been established in such large cities as Beijing or Shanghai. Nonetheless, the central-provincial administrative division in China leads to asymmetrical execution according to the Institutional Theory. Urban systems have the advantage of concentrated resources, concentration of policies, whereas rural areas lacks staff with qualified personnel, infrastructural problems and decentralized funding. These institutional constraints translate the resource investments into efficiency gains (Figure 4).

### Strategic integration of lessons from Japan and China

A mixed Resource-Based and Institutional Theory viewpoint implies that the efficiency in older adult care would be ideal in the case of sufficient financial, human, and technological resources when they are being used within the institutional frameworks that would have the capability to coordinate and maintain them. The most important lesson to be learned, with the example of Japan, is that national LTCI system can guarantee stable funding, trained workforce provision, and highly developed infrastructure but only in case local administrative potential is high. The lesson in China is that strategically chosen and heavily invested city resources, such as digital platforms and facility expansion, can reach a high efficiency very fast, yet supportive governance needs to expand to rural and underserved regions.

The combination of these strengths implies the development of structures of national uniformity in terms of coverage and quality of services (Institutional Theory) and the strategic distribution of resources to address local issues of demography and geography (Resource-Based Theory).

This table compares key strengths of older adult care systems in Japan and China and proposes combined strategies for scalable global application (Table 3).

**Table 3:** Integrated older adult care strategy framework.

Strategic axis	Japan (strength)	China (strength)	Recommendation
Service structure	Universal LTCI + CBICS	Pilot LTCI + community-based trials	Integrate national coverage with pilots
Technology use	Advanced robotics and ICT (urban)	AI + telemedicine for rural outreach	Blend AI scalability with robotics
Financial mechanism	Multi-tier funding (tax + premiums)	Government-led + local co-financing	Cross-national funding models
Preventive care	Community prevention for healthy older adult	Limited but emerging pilot programs	Expand preventive education regionally

### Interpretation of findings

#### How efficiency translates to improved health outcomes

The results highlight that the efficiency of care in old age is enhanced through the strategic convergence of the resources available, including funding, workforce, and infrastructure, with institutional arrangements that provide equal and equal access to the services.

Physical health: Increases in life expectancy and management of chronic diseases in the two countries have been linked to investments in resources-medical personnel, and modernization of facilities, which have been achieved via stable policy structures.

Mental health: Community-based models of care in Japan and urban China promote better psychological well-being when resourceful and institutionally supported.

Social health: High social engagement is realized when services operate in community networks and that are supported by funding and administration coordination.

**Figure 5:**
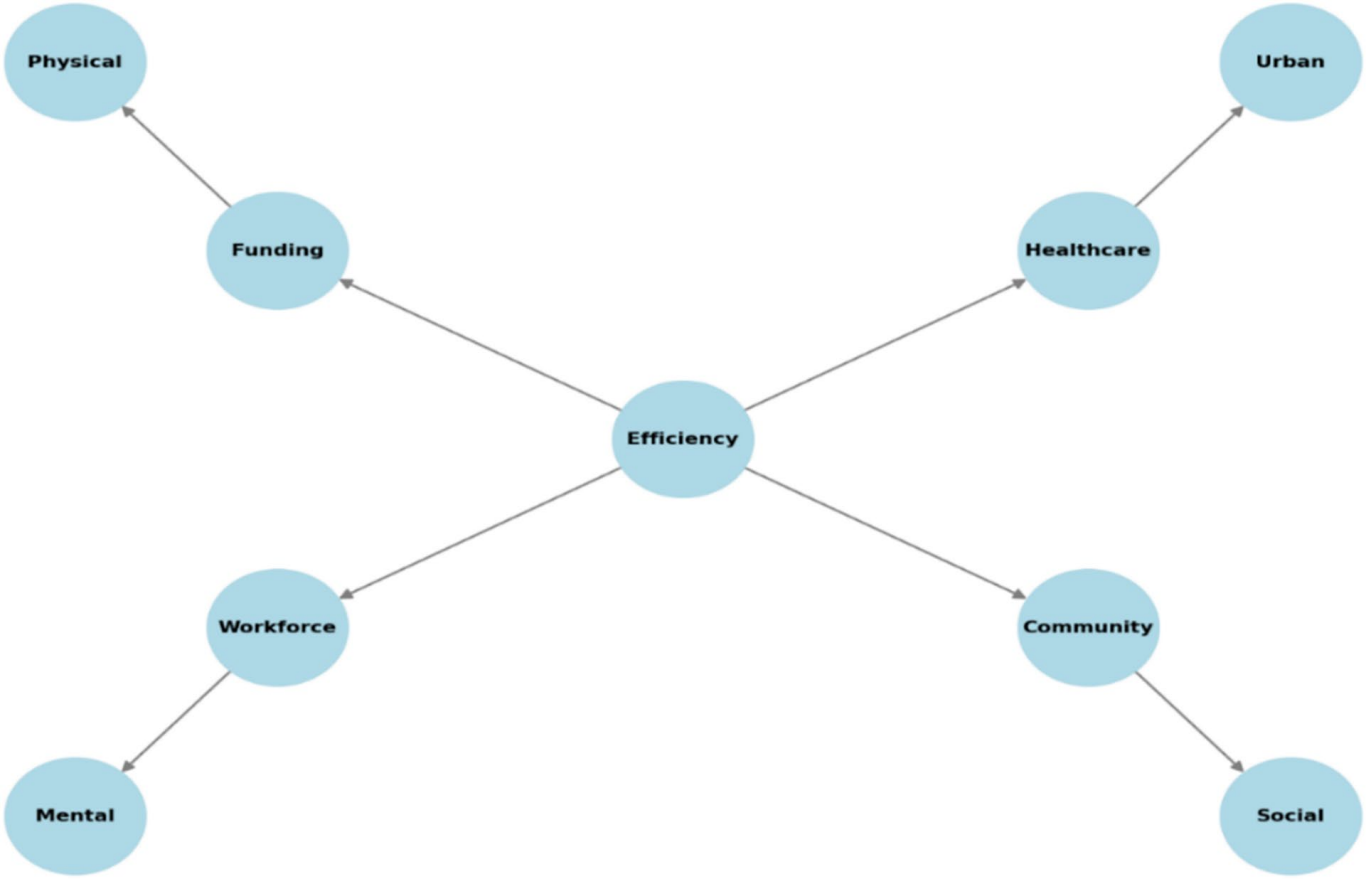
Chart illustrating the impact of efficiency measures on health outcomes.

The differing outcome between China and Japan can be said to be a contrast between resource sufficiency (Resource-Based Theory) and the structure of governance (Institutional Theory). National standards and resource sufficiency, both of which foster efficiency, support the universal LTCI in Japan, but not the non-standardized provincial execution in China, which hinders the urban health progress and urban health gains in the context of uneven convergence across the country (Figure 5).

The chart displays how enhanced older adult care production results in superior health results through an effective combination of important factors. Through its four core components “Efficiency” acts as the central driving force which enhances the healthcare system at the Los Angeles Center. The combination of funding and workforce development advances physical health while healthcare modernization enhances urban healthcare benefits together with community-based social programs that enhance overall social health. A well-structured older adult care system creates better overall well-being because each factor intertwines with the others according to the diagram.

## Policy implications

The results of the present study suggest that enhancing efficiency in older adult care requires the possibility of mobilizing and maintaining financial, human, and technological resources in the framework of strong institutions. In the case of China, what is most pressing is to increase the flow of skilled personnel, especially within rural and underserved regions. The solution to the workforce shortage would be to increase caregiver training programs that incorporate both medical and person-centered care skills. Simultaneously, the government ought to hike dedicated investments toward building rural facilities and expanding telemedicine, with the specifications of the infrastructure being equal to what the cities receive. The investments alone can only reflect substantial efficiency gains when they are put in action with enhanced institutional coordination between central and provincial governments so that there is a lessening of the gap in the implementation of policies.

Japan is a country with different policy priorities owing to a high resource capacity already yet with strains on how to remain efficient, given its demographic and fiscal pressure. Japan must also assess its efficiency in measuring social health to ensure that it is leading in the provision of older adult care by integrating indicators of isolation and involvement of the community members in its continuous evaluation of efficiency. This would mean that the quality of care would show physical and mental well-being but also social well-being. Moreover, technology-based solutions, such as robotic caregivers and remote monitoring systems, should be given more attention to alleviate issues with workforce shortages. Nevertheless, finance must be provided at the municipality level through the financial support of these technologies to prevent extending the gap between the well-financed and under-financed localities.

In a more global sense, the lessons of these two countries act as a scalable model of aging societies on the planet. The Resource-Based Theory postulates that efficiency is bound to the wise utilization of financial, workforce, and technological resources, whereas the Institutional Theory pays attention to the existence of governance structures, which are to coordinate the aforementioned resources in a fair manner. Countries that are currently experiencing rapid demographic transitions can use the experience of Japan in terms of creating standardized national care models that would assure the consistency of service provision, and China with its creative AI and digital technology implementations that would widen access to care in resource-constrained environments. By coordinating these solutions, policy makers can develop older adult care systems that are efficient and flexibility which translates to provision of sustainable health outcomes under social, and economic, varying environments.

## Conclusion

This study proves that the efficiency of older adult care in China and Japan depends on the interaction between the availability of resources and institutional organization. Based on the Resource-Based Theory, the research results indicate that sufficient financial resources that are strategically used, availability of well-trained and accessible workforce, and having a modern infrastructure are important aspects in recording positive physical, mental, and social health outcomes in older adult. Institutional Theory adds to this view by explaining how governance systems, policy systems and administrative capabilities shape the manner in which these resources are distributed, synchronized and maintained over the duration of time.

Universal Long-Term Care Insurance system used in Japan, combined with local-level implementation and rigorous preventive care programs has allowed the country to achieve high efficiency and health outcomes across the population consistently. Nevertheless, it will be necessary to invest in technological innovation and integration of social well-being metrics into performance measurement in order to retain these achievements amid the challenges of increased costs and workforce scarcity. China, by contrast, has experienced considerable returns in the urban centers as a result of strategic investment in workforce education, expansion of facilities, and digital platforms of health. However, efficiency and health performance in rural areas are still limited due to a lack of investment and health policy implementation imbalance, thus requiring better central-provincial cooperation and more equal resource allocation.

In the comparative analysis, efficiency is not only contributed by the availability of resources but the institutional capability to utilize the available resources. Policymakers in other aging societies can learn complementary lessons in China and Japan, where nationally standardized care and technology-driven outreach, respectively, have led to quality and consistency and the ability to increase access in settings where resources are scarce.

By integrating Resource-Based Theory with Institutional Theory, researchers can apply a theory-driven strategy within a cross-national, longitudinal framework. This approach provides a pathway toward developing fair care systems that address the health and social needs of aging populations in both developed urban centers and underserved rural communities.

## Data Availability

The original contributions presented in the study are included in the article/supplementary material, further inquiries can be directed to the corresponding author.
